# Change in Colorectal Cancer Tests Submitted for Reimbursement in Switzerland 2012–2018: Evidence from Claims Data of a Large Insurance

**DOI:** 10.3389/ijph.2021.1604073

**Published:** 2021-10-28

**Authors:** Rémi Schneider, Markus Näpflin, Lamprini Syrogiannouli, Sarah Bissig, Kali Tal, Jean-Luc Bulliard, Cyril Ducros, Oliver Senn, Kevin Selby, Caroline Bähler, Eva Blozik, Reto Auer

**Affiliations:** ^1^ Berner Institut für Hausarztmedizin, Medizinische Fakultät, Universität Bern, Bern, Switzerland; ^2^ Department of Health Sciences, Helsana Insurance Group, Zürich, Switzerland; ^3^ Center for Primary Care and Public Health, University of Lausanne, Lausanne, Switzerland; ^4^ Institut für Hausarztmedizin, Universitätsklinikum Zürich, Zürich, Switzerland

**Keywords:** colorectal cancer screening, fecal occult blood test, colonoscopy, testing rates, Swiss health insurance

## Abstract

**Objectives:** Guidelines recommend colorectal cancer (CRC) screening by fecal occult blood test (FOBT) or colonoscopy. In 2013, Switzerland introduced reimbursement of CRC screening by mandatory health insurance for 50-69-years-olds, after they met their deductible. We hypothesized that the 2013 reimbursement policy increased testing rate.

**Methods:** In claims data from a Swiss insurance, we determined yearly CRC testing rate among 50-75-year-olds (2012–2018) and the association with socio-demographic, insurance-, and health-related covariates with multivariate-adjusted logistic regression models. We tested for interaction of age (50–69/70–75) on testing rate over time.

**Results:** Among insurees (2012:355′683; 2018:348′526), yearly CRC testing rate increased from 2012 to 2018 (overall: 8.1–9.9%; colonoscopy: 5.0–7.6%; FOBT: 3.1–2.3%). Odds ratio (OR) were higher for 70–75-year-olds (2012: 1.16, 95%CI 1.13–1.20; 2018: 1.05, 95%CI 1.02–1.08). Deductible interacted with changes in testing rate over time (*p* < 0.001). The increase in testing rate was proportionally higher among 50-69-years-olds than 70-75-year-olds over the years.

**Conclusions:** CRC testing rate in Switzerland increased from 2012 to 2018, particularly among 50-69-years-olds, the target population of the 2013 law. Future studies should explore the effect of encouraging FOBT or waiving deductible.

## Introduction

Colorectal cancer (CRC) is the third leading cause of cancer mortality in Switzerland, killing 1600 people annually [1]. CRC screening can reduce CRC mortality [2–4], so the US Preventive Services Task Force recommend screening average-risk patients aged 50–75 years with either colonoscopy every 10 years or fecal occult blood test (FOBT) every one to 2 years, in line with other international recommendations [4–8]. CRC screening seems to be underused in Switzerland but the only nationwide data are from the Swiss Health Interview Survey (SHIS), a survey of Swiss inhabitants conducted every 5 years, with percentages weighted to represent the whole population. In 2012, 39.5% of the 50–75 years-old had reported to be tested for CRC within the recommended interval (32.8% had a colonoscopy and 13.2% an FOBT) [9]. Studies conducted in selected Swiss subpopulations between 2005 and 2017 showed a proportion varying between 33.6 and 49%, but these studies estimated the cumulative number of participants tested over a long period and could not identify short-term changes in the testing rate [10–13].

In Switzerland, mandatory basic health insurance offers a range of deductibles (cost-sharing model). The monthly premium can be reduced by choosing a high deductible plan (HDHP) or opting for a managed-care organisation model [Health Maintenance Organization (HMO), family physician, or telemedicine entry point]. Bills are either paid directly by the insuree (*tiers payant*) who then requests reimbursement from the insurance or are paid directly by the insurance (*tiers garant*), which then checks the bill and charges the insuree the co-payment. In July of 2013, mandatory health insurance was legally required to reimburse preventive screening tests for 50-69-year-olds. Before that, insurances reimbursed only diagnostic CRC testing and this reimbursement was not excluded from the yearly deductible. There is no reimbursement for CRC screening in other age groups. FOBT is much less expensive (about 9 CHF; 8.4€) than colonoscopy (about 800–1600 CHF; 750–1500 €) [14].

Factors that affect CRC screening rate in Switzerland are still unclear. An analysis of the SHIS found that 70–75 years-olds were more likely to be tested in 2012 than 50–69 years-old [9]. Insurance types are also an important determinant of CRC screening [15–17]. In the 2012 SHIS, respondents with HDHP and basic insurance were up to 20% less likely to have been tested for CRC by colonoscopy than those with low deductible health plan (LDHP) and private insurance, but no such difference was evident for FOBT [9]. The influence of gender, education or income on CRC testing rate in Switzerland is not clearly established [10], but low income is associated with forgoing health care [18].

We sought to characterize the changes in CRC screening rate in Switzerland after reimbursement became law in 2013. We thus set out to determine if yearly CRC testing rate among insures aged 50–69 increased after the law, and then compared it to testing rate among those aged 70–75. We also assessed if the law increased CRC testing more among those with LDHP than HDHP policies, given that the law did not waive deductible. We hypothesized that the reimbursement raised overall the CRC testing rate among those aged 50–69 and those with LDHP. The real number of CRC tests performed in Switzerland is unknown. We chose to use claims data from a large health insurance as a surrogate to estimate it. We used the number of claims for reimbursement for CRC tests to estimate overall testing rates and detect changes in colonoscopy and FOBT rates within and between health plans after changes in reimbursement policy [19–23].

## Methods

### Study Design, Data Source and Participants

We retrospectively analyzed yearly claims data (January 1st, 2012 to December 31st, 2018) provided by Helsana Insurance Group of 50–75 years-old Swiss residents with mandatory basic health insurance. We performed repeated yearly cross-sectional analyses of this data. Helsana Insurance covers about 1.2 million insures, i.e., about 15% of the Swiss population, representing the general population in all swiss cantons [24].

Helsana Group collects data from the billing claims of each insuree for reimbursement of medical treatments and services. Billing for which no reimbursement has been claimed (e.g., in the “tiers payant” reimbursement process) are thus not available in the dataset. Personal information is collected during the conclusion of insurance contract. All data used in this study were extracted from Helsana’s database. Three authors (RS, RA and LS) specified the procedure for the analysis and variable definitions. The Helsana research team specified data management procedures, variable definition and the procedure for data analysis (MN, EB, CB). Data were retrospective, de-identified and anonymized so ethical approval was not required under the Swiss Human Research Act.

For each index year from 2012 to 2018, we included insurees aged ≥50 on January 1st and ≤75 on December 31st who had been continuously insured over the course of the year. We excluded insurees who died or who left Helsana during the index year. Insurees identified as having inflammatory bowel disease (IBD) the year previous to the study year were also excluded based on a previous established procedure [25].

### Outcomes and Covariates

Our primary outcome of interest was annual claimed CRC testing rate per insured population (2012–2018). We defined CRC testing rate as the proportion (percentage) of insurees who claimed at least one CRC test during the index calendar year. To detect all CRC tests, we identified billing codes for colonoscopies, sigmoidoscopies, and polyp removal in the Swiss Diagnosis-Related Groups (DRGs), the Swiss Classification of Surgical Operation (CHOP), the Swiss Ambulatory Procedures codes (TARMED), and codes for FOBT in the Swiss Analysis List of laboratory measures (AL). We include the list of billing codes in the Supplementary File S1. Codes do not differentiate between guaiac-based FOBTs (gFOBT) and immunological FOBTs (FIT); they were billed under the same code (1583). The billing codes also do not distinguish between diagnostic and screening tests, so we could only calculate the CRC testing rate rather than the screening rate. Secondary outcome was the rate at which insurees were tested with colonoscopy and/or FOBT.

To adjust our analysis for factors that might influence CRC testing, we extracted several data about sociodemographic, insurance, and health factors. All the covariates were collected by Helsana Group. We chose covariates based on a review of the literature.

The sociodemographic factors for adjustment included age, gender, and community of residence (urban, intermediate or rural, as defined by the Federal Statistical Office). We extracted information about participation in a managed care model (family doctor, HMO, or telemedicine model) and monthly level of deductible. To assess chronic health conditions, we used an updated measure of the Pharmacy-based Cost Group (PCG) [26]. Billing data in outpatient settings in Switzerland do not contain clinical diagnoses, but they do mention medications. The PCG model uses medications billing to distinguish between 21 chronic health conditions and has been used with similar algorithms as a proxy for health status [27–30].

### Statistical Analyses

We first used descriptive statistics to establish the baseline characteristics of the study population. We then extracted the number of insurees who claimed reimbursement for a colonoscopy or FOBT and calculated the percentage to determine the testing rate in the overall population and for each strata of covariates. We then calculated the difference of testing rates between the oldest (70–75) and youngest (50–69) insurees and those with LDHP and HDHP in 2012 and 2018 (absolute difference). We also computed the ratio of the difference between testing rates over the testing rate of the oldest or those with LDHP (relative difference). For each year, we used the data of insurees for that year and did not track insurees over the years. As done previously [31], we grouped sigmoidoscopies and colonoscopies together, since sigmoidoscopies are rare in Switzerland. We merged interventions performed in inpatient- and outpatient-settings to calculate overall colonoscopy testing rate. We included insurees who had both a colonoscopy and an FOBT tests in the FOBT group because we assumed that positive FOBT results led to a colonoscopy. We grouped insurees into two age categories (50–69 vs. 70–75). We subdivided insurees into four categories of deductible (low: 300 CHF; medium low: 500; medium high: 1000 or 1500 CHF; and, high: 2000 or 2500 CHF).

We fitted two logistic regression models to estimate the odds ratio (OR) of CRC testing with 95% confidence interval (95% CI). The first was a binomial multivariate-adjusted logistic regression model comparing insurees who had and had not been tested for CRC. The second was a multinomial multivariate-adjusted logistic regression model that compared the proportion of insurees tested for each type of exam to those who had not been tested. In both statistical models, “no testing” was the baseline category. We adjusted for deductible, age, gender, urban status of the community of residence, managed care model, and number of PCG in the previous year. We used these two models to evaluate the association between any test and our covariates. We further tested for the interaction between year of testing and age category on CRC testing through multivariate adjusted regression. In a multivariate adjusted logistic regression model including all participants across the years, we tested the statistical significance of the interaction term “year: age category”, controlling for each individual variable such as sex, deductible, managed care status, location and year and age category. We tested then the interaction between year of testing and deductible category using the interaction term “year: deductible category”. We calculated the *p*-value with the Wald test.

We performed sensitivity analyses to determine if other factors or covariates influenced our results. To check for the possible influence of screening programs in Vaud (launched in 2015) and Uri (launched in 2014) on the overall CRC testing rates in Switzerland, we repeated the analysis after excluding insurees who lived in those cantons.

The threshold for statistical significance for all analyses was *p* < 0.05. We used R Software for all statistical analyses.

## Results

From the dataset of Helsana Group, we included about 350′000 insurees in the analysis for each year. The characteristics of the population for 2012, 2015, and 2018 are described in Table 1. Characteristics of insurees were overall comparable over the years but indicated a growing proportion of those opting for a high deductible (2000/2500) or a managed care plan.

**TABLE 1:** Characteristics of 50–75 years-old insurees over the full year in 2012, 2015 and 2018, Helsana Database, Switzerland.

Variable	2012	2015	2018
Total participants, N	355 683	360 308	348 526
Socio-demographics			
Age in years			
50–69, N (col %)	289 118 (81.3%)	288 634 (80.1%)	275 860 (79.2%)
70–75, N (col %)	66 565 (18.7%)	71 674 (19.9%)	72 666 (20.8%)
Gender (Male), N (%)	168 898 (47.5%)	172 062 (47.8%)	166 466 (47.8%)
Residence			
Urban, N (col %)	97 069 (27.3%)	97 642 (27.1%)	95 745 (27.5%)
Intermediate, N (col %)	174 301 (49.0%)	177 147 (49.2%)	169 046 (48.5%)
Rural, N (col %)	84 313 (23.7%)	85 519 (23.7%)	83 735 (24.0%)
Insurance plan			
Deductible (CHF)			
300, N (col %)	189 967 (53.4%)	197 489 (54.8%)	192 335 (55.2%)
500, N (col %)	80 521 (22.6%)	72 513 (20.1%)	62 517 (17.9%)
1000/1500, N (col %)	45 956 (12.9%)	41 928 (11.6%)	36 306 (10.4%)
2000/2500, N (col %)	39 239 (11.0%)	48 378 (13.4%)	57 368 (16.5%)
Managed Care (yes), N (%)	154 850 (43.5%)	193 956 (53.8%)	213 044 (61.1%)
Health status proxies			
Number of PCG groups^a^			
None, N (col %)	157 722 (44.3%)	152 772 (42.4%)	142 002 (40.7%)
1, N (col %)	69 289 (19.5%)	67 122 (18.6%)	66 522 (19.1%)
2, N (col %)	54 880 (15.4%)	54 574 (15.1%)	53 464 (15.3%)
≥3, N (col %)	73 792 (20.7%)	85 840 (23.8%)	86 538 (24.8%)

a Pharmacy-based Cost Group [26], based on data of the previous year.

Percentages refer to the proportion of the population (50–75 years old) of the studied calendar year.

CHF, Swiss Franc.

**FIGURE 1:**
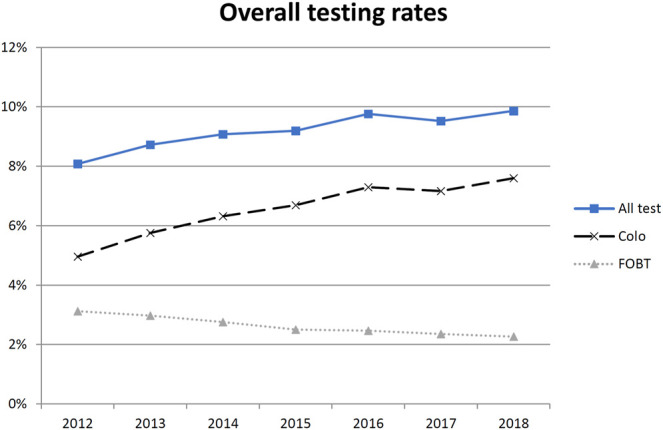
Percentages of 50–75 years-old insurees who underwent colonoscopy (dashed line), fecal occult blood test (dotted line) or both (solid line), Helsana Database, Switzerland, January 2012 to December 2018. FOBT, fecal occult blood test.

Testing rates for claimed colonoscopy and FOBT on average in the study population and by covariates are found in Figure 1 and Table 2 and Supplementary File S3. Overall annual CRC testing rate increased over time from 8.1% (95%CI: 8.0–8.2) per year in 2012 to 9.9% (95%CI: 9.8–10.0) in 2018 (*p*-value for trend across year <0.01). The colonoscopy rate increased from 5.0% (95%CI: 4.9–5.0) in 2012 to 7.6% (95%CI: 7.5–7.7) in 2018; the FOBT rate decreased from 3.1% (95%CI: 3.1–3.2) to 2.3% (95%CI: 2.2–2.3). .

**TABLE 2:** numbers of 50–75 years-old insurees who underwent overall colorectal cancer testing (colonoscopy and/or fecal occult blood test), colonoscopy or fecal occult blood test in 2012, 2015 and 2018, Helsana Database, Switzerland (data for the remaining years are available in the Supplementary File S3).

Variable	Overall			Colonoscopy			FOBT/both		
	2012	2015	2018	2012	2015	2018	2012	2015	2018
Population N, %, (95% CI)	28 731	33 126	34 378	17 630	24 110	26 483	11 101	9 016	7 895
	8.1	9.2	9.9	5.0	6.7	7.6	3.1	2.5	2.3
	(8.0–8.2)	(9.1–9.3)	(9.8–10.0)	(4.9–5.0)	(6.6–6.8)	(7.5–7.7)	(3.1–3.2)	(2.5–2.6)	(2.2–2.3)
Age in years									
50–69 N, %, (95% CI)	22 096	25 192	26 204	13 754	18 622	20 482	8 342	6 570	5 722
	7.6	8.7	9.5	4.8	6.5	7.4	2.9	2.3	2.1
	(7.5–7.7)	(8.6–8.8)	(9.4–9.6)	(4.7–4.8)	(6.4–6.5)	(7.3–7.5)	(2.8–2.9)	(2.2–2.3)	(2.0–2.1)
70–75 N, %, (95% CI)	6 635	7 934	8 174	3 876	5 488	6 001	2 759	2 446	2 173
	10.0	11.1	11.2	5.8	7.7	8.3	4.1	3.4	3.0
	(9.7–10.2)	(10.8–11.3)	(11.0–11.5)	(5.6–6.0)	(7.5–7.9)	(8.1–8.5)	(4.0–4.3)	(3.3–3.6)	(2.9–3.1)
Gender									
Female N, %, (95% CI)	13 896	15 999	16 587	9 356	12 655	13 700	5 479	4 472	4 091
	8.2	9.3	10.0	5.0	6.7	7.5	2.9	2.4	2.2
	(8.1–8.4)	(9.2–9.4)	(9.8–10.1)	(4.9–5.1)	(6.6–6.8)	(7.4–7.7)	(2.9–3.0)	(2.3–2.4)	(2.2–2.3)
Male N, %, (95% CI)	14 835	17 127	17 791	8 274	11 455	12 783	5 622	4 544	3 804
	7.9	9.1	9.8	4.9	6.7	7.7	3.3	2.6	2.3
	(7.8–8.1)	(9.0–9.2)	(9.6–9.9)	(4.8–5.0)	(6.5–6.8)	(7.5–7.8)	(3.2–3.4)	(2.6–2.7)	(2.2–2.4)
Residence									
Urban N, %, (95% CI)	7 705	8 958	9 378	4 824	6 712	7 296	2 881	2 246	2 082
	7.9	9.2	9.8	5.0	6.9	7.6	3.0	2.3	2.2
	(7.8–8.1)	(9.0–9.4)	(9.6–10.0)	(4.8–5.1)	(6.7–7.0)	(7.4–7.8)	(2.9–3.1)	(2.2–2.4)	(2.1–2.3)
Intermediate N, %, (95% CI)	14 811	17 195	17 296	9 059	12 479	13 365	5 752	4 716	3 931
	8.5	9.7	10.2	5.2	7.0	7.9	3.3	2.7	2.3
	(8.4–8.6)	(9.6–9.9)	(10.1–10.4)	(5.1–5.3)	(6.9–7.2)	(7.8–8.0)	(3.2–3.4)	(2.6–2.7)	(2.3–2.4)
Rural N, %, (95% CI)	6 215	6 973	7 704	3 747	4 919	5 822	2 468	2 054	1 882
	7.4	8.2	9.2	4.4	5.8	7.0	2.9	2.4	2.2
	(7.2–7.6)	(8.0–8.3)	(9.0–9.4)	(4.3–4.6)	(5.6–5.9)	(6.8–7.1)	(2.8–3.0)	(2.3–2.5)	(2.1–2.4)
Deductible (CHF)									
300 N, %, (95% CI)	17 548	20 709	21 343	10 800	15 121	16 441	6 748	5 588	4 902
	9.2	10.5	11.1	5.7	7.7	8.5	3.6	2.8	2.5
	(9.1–9.4)	(10.3–10.6)	(10.9–11.2)	(5.6–5.8)	(7.5–7.8)	(8.4–8.7)	(3.5–3.6)	(2.8–2.9)	(2.5–2.6)
500 N, %, (95% CI)	6 566	6 663	6 466	3 965	4 752	4 840	2 601	1 911	1 626
	8.2	9.2	10.3	4.9	6.6	7.7	3.2	2.6	2.6
	(8.0–8.4)	(9.0–9.4)	(10.1–10.6)	(4.8–5.1)	(6.4–6.7)	(7.5–8.0)	(3.1–3.4)	(2.5–2.8)	(2.5–2.7)
1000/1500 N, %, (95% CI)	2 801	2 974	2 901	1 736	2 183	2 291	1 065	791	610
	6.1	7.1	8.0	3.8	5.2	6.3	2.3	1.9	1.7
	(5.9–6.3)	(6.8–7.4)	(7.7–8.3)	(3.6–4.0)	(5.0–5.4)	(6.1–6.6)	(2.2–2.5)	(1.8–2.0)	(1.5–1.8)
2000/2500 N, %, (95% CI)	1 816	2 780	3 668	1 129	2 054	2 911	687	726	757
	4.6	5.7	6.4	2.9	4.2	5.1	1.8	1.5	1.3
	(4.4–4.8)	(5.5–6.0)	(6.2–6.6)	(2.7–3.1)	(4.1–4.4)	(4.9–5.3)	(1.6–1.9)	(1.4–1.6)	(1.2–1.4)
Managed care									
No N, %, (95% CI)	16 224	15 322	13 262	10 098	11 205	10 286	6 126	4 117	2 976
	8.1	9.2	9.8	5.0	6.7	7.6	3.1	2.5	2.2
	(8.0–8.2)	(9.1–9.4)	(9.6–10.0)	(4.9–5.1)	(6.6–6.9)	(7.4–7.7)	(3.0–3.1)	(2.4–2.6)	(2.1–2.3)
Yes N, %, (95% CI)	12 507	17 804	21 116	7 532	12 905	16 197	4 975	4 899	4 919
	8.1	9.2	9.9	4.9	6.7	7.6	3.2	2.5	2.3
	(7.9–8.2)	(9.0–9.3)	(9.8–10.0)	(4.8–5.0)	(6.5–6.8)	(7.5–7.7)	(3.1–3.3)	(2.5–2.6)	(2.2–2.4)
Number of PCG^a^									
None N, %, (95% CI)	9 395	10 665	10 688	5 823	7 960	8 409	3 572	2 705	2 279
	6.0	7.0	7.5	3.7	5.2	5.9	2.3	1.8	1.6
	(5.8–6.1)	(6.8–7.1)	(7.4–7.7)	(3.6–3.8)	(5.1–5.3)	(5.8–6.0)	(2.2–2.3)	(1.7–1.8)	(1.5–1.7)
1 N, %, (95% CI)	6 166	6 514	6 830	3 658	4 684	5 222	2 508	1 830	1 608
	8.9	9.7	10.3	5.3	7.0	7.9	3.6	2.7	2.4
	(8.7–9.1)	(9.5–9.9)	(10.0–10.5)	(5.1–5.5)	(6.8–7.2)	(7.6–8.1)	(3.5–3.8)	(2.6–2.9)	(2.3–2.5)
2 N, %, (95% CI)	5 304	5 843	6 069	3 126	4 124	4 648	2 178	1 719	1 421
	9.7	10.7	11.4	5.7	7.6	8.7	4.0	3.1	2.7
	(9.4–9.9)	(10.4–11.0)	(11.1–11.6)	(5.5–5.9)	(7.3–7.8)	(8.4–8.9)	(3.8–4.1)	(3.0–3.3)	(2.5–2.8)
≥3 N, %, (95% CI)	7 866	10 104	10 791	5 023	7 342	8 204	2 843	2 762	2 587
	10.7	11.8	12.5	6.8	8.6	9.5	3.9	3.2	3.0
	(10.4–10.9)	(11.5–12.0)	(12.2–12.7)	(6.6–7.0)	(8.4–8.8)	(9.3–9.7)	(3.7–4.0)	(3.1–3.3)	(2.9–3.1)

a Pharmacy-based Cost Group [26], based on data of the previous year.

Percentages refer to the proportion of insurees which were tested in the subgroup of the studied population, with the 95% confidence interval.

CHF, Swiss Franc; FOBT, fecal occult blood test.

**FIGURE 2:**
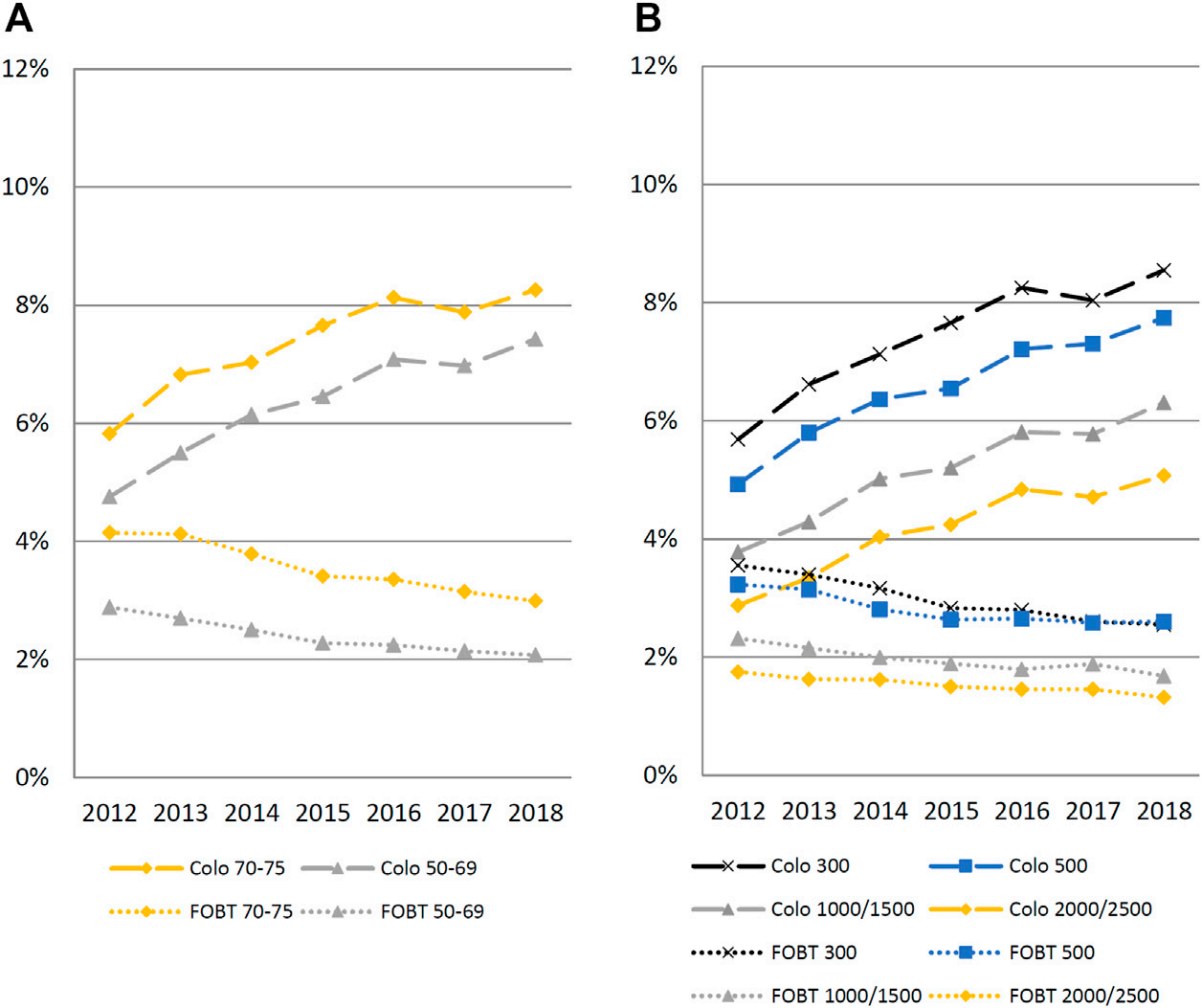
Percentages of 50–75 years-old insurees who underwent colonoscopy (dashed line) or fecal occult blood test (dotted line), by age and deductible categories, Helsana Database, Switzerland, January 2012 to December 2018. Panel **(A)** by age; Panel **(B)** by deductible. FOBT, fecal occult blood test. Interaction term for age: 70–75 over time: 0.978 (0.973 0 0.984); *p* < 0.001. Interaction term for deductible: 500 CHF: 1.009 (1.003–1.015); for 1000/1500 CHF 1.018 (1.010–1.026); 2000/2500 CHF 1.026 (1.018–1.034); *p* < 0.001.

Overall CRC testing rate increased in insurees aged 50–69 from 7.6% (95%CI: 7.5–7.7) in 2012 to 9.5% (95%CI: 9.4–9.6) in 2018; and in insurees aged 70–75 from 10.0% (95%CI: 9.7–10.2) to 11.2% (95%CI: 11.0–11.5) (*p*-value for trend across years <0.01 for both age groups). Insurees aged 70–75 years had the highest overall testing rate, colonoscopy rate and FOBT rate in 2012, 2015, and 2018. The absolute difference in CRC testing rates between age categories decreased over time. (Table 2 and Figure 2A; Supplementary File S3 for remaining years).

Overall CRC testing rate increased by insurees with LDHP (300 CHF) from 9.2% (95%CI: 9.1–9.4) in 2012 to 11.1% (95%CI: 10.9–11.2) in 2018; and by insurees with HDHP (2000/2500 CHF) from 4.6% (95%CI: 4.4–4.8) to 6.4% (95%CI: 6.2–6.6). The absolute difference in overall testing rate between deductible categories stayed almost unchanged over the years. The absolute difference in colonoscopy rate between LDHP and HDHP categories increased over time (from 2.8% in 2012 to 3.5% in 2018) and decreased over time for FOBT (from 1.8% in 2012 to 1.2%). (Table 2 and Figure 2B).

The adjusted odds ratios of having been tested with any CRC test are listed in Table 3 and in Supplementary File S4. Covariates significantly associated with higher CRC testing rate were: older age, low deductible, male gender, residence in an intermediate urban community, participation in a managed-care model and presenting chronic health conditions. These results were similar for each index year.

**TABLE 3:** Adjusted odds ratios of 50–75 years-old insurees who underwent any colorectal cancer test, in 2012, 2015 and 2018, Helsana Database, Switzerland (data for the remaining years are available in the Supplementary File S4).

	2012	2015	2018
	OR (CI 95%)	OR (CI 95%)	OR (CI 95%)
Age in years (ref: 50–69)			
70–75	1.16 (1.13–1.20)*	1.14 (1.11–1.17)*	1.05 (1.02–1.08)*
Gender (ref: male)			
Female	0.92 (0.90–0.94)*	0.93 (0.91–0.95)*	0.93 (0.91–0.96)*
Residence (ref: urban)			
intermediate	1.09 (1.06–1.13)*	1.08 (1.05–1.11)*	1.07 (1.04–1.10)*
rural	0.95 (0.92–0.98)*	0.90 (0.87–0.93)*	0.96 (0.93–0.99)*
Deductible (CHF) (ref: 300)			
500	0.91 (0.89–0.94)*	0.90 (0.87–0.92)*	0.96 (0.93–0.98)*
1000/1500	0.75 (0.72–0.78)*	0.75 (0.72–0.78)*	0.79 (0.76–0.82)*
2000/2500	0.59 (0.56–0.62)*	0.63 (0.60–0.65)*	0.65 (0.63–0.68)*
Managed care (ref: no)			
Yes	1.09 (1.06–1.12)*	1.07 (1.05–1.10)*	1.08 (1.06–1.11)*
Number of PCG^a^ (ref: no)			
1	1.42 (1.37–1.47)*	1.32 (1.28–1.37)*	1.30 (1.26–1.34)*
2	1.49 (1.44–1.55)*	1.41 (1.36–1.46)*	1.40 (1.36–1.45)*
≥3	1.63 (1.57–1.68)*	1.53 (1.48–1.58)*	1.53 (1.48–1.57)*

a Pharmacy-based Cost Group [26], based on data of the previous year.

*Confidence intervals do not cross the OR of 1.

All the odds ratios are adjusted for the other covariates, in a binomial logistic regression model.

CHF, Swiss Franc.

Table 4 summarizes the odds ratios of having been tested with colonoscopy or FOBT in 2012, 2015, and 2018 from multivariate adjusted multinomial regression models (data for remaining years in Supplementary File S5). The covariates associated throughout the years with having been tested with colonoscopy were low deductible, residence in an intermediate urban community, participation in a managed-care model, and presenting chronic health conditions. The probability of having been tested with a colonoscopy was higher in insurees aged 70–75 years in 2012, 2015, but not in 2018, and higher in men in 2015 and 2018, but not in 2012. Covariates associated with FOBT testing were older age, low deductible, male gender, residence in an intermediate urban community, participation in a managed-care model, and presenting chronic health conditions. The results were similar after excluding the insurees living in Vaud and Uri (data not shown).

**TABLE 4:** Multinomial logistic regression model, adjusted odds ratios of 50–75 years-old insurees who underwent colonoscopy or fecal occult blood test, in 2012, 2015 and 2018, Helsana Database, Switzerland (data for the remaining years are available in the Supplementary File S5).

	Test	2012	2015	2018
		OR (CI 95%)	OR (CI 95%)	OR (CI 95%)
Age in years (ref: 50–69)				
70–75	Colo	1.08 (1.04–1.12)*	1.07 (1.03–1.10)*	0.99 (0.96–1.02)
	FOBT	1.30 (1.24–1.36)*	1.34 (1.27–1.40)*	1.26 (1.20–1.33)*
Gender (ref: male)				
Female	Colo	0.97 (0.94–1.00)	0.96 (0.93–0.98)*	0.94 (0.91–0.96)*
	FOBT	0.84 (0.80–0.87)*	0.85 (0.82–0.89)*	0.93 (0.89–0.97)*
Residence (ref: urban)				
intermediate	Colo	1.07 (1.04–1.11)*	1.05 (1.01–1.08)*	1.06 (1.03–1.09)*
	FOBT	1.13 (1.08–1.18)*	1.17 (1.12–1.24)*	1.09 (1.03–1.15)*
rural	Colo	0.92 (0.88–0.96)*	0.85 (0.82–0.88)*	0.93 (0.90–0.97)*
	FOBT	1.00 (0.95–1.06)	1.06 (1.00–1.12)	1.06 (0.99–1.12)
Deductible (CHF) (ref: 300)				
500	Colo	0.90 (0.87–0.93)*	0.87 (0.85–0.90)*	0.93 (0.90–0.96)*
	FOBT	0.93 (0.89–0.98)*	0.96 (0.91–1.01)	1.05 (1.00–1.12)
1000/1500	Colo	0.76 (0.72–0.80)*	0.75 (0.71–0.78)*	0.80 (0.77–0.84)*
	FOBT	0.73 (0.68–0.78)*	0.76 (0.70–0.82)*	0.75 (0.68–0.81)*
2000/2500	Colo	0.60 (0.56–0.64)*	0.62 (0.59–0.65)*	0.66 (0.63–0.69)*
	FOBT	0.57 (0.52–0.62)*	0.63 (0.58–0.69)*	0.61 (0.57–0.67)*
Managed care (ref: no)				
Yes	Colo	1.06 (1.02–1.09)*	1.06 (1.03–1.09)*	1.07 (1.04–1.10)*
	FOBT	1.15 (1.10–1.19)*	1.11 (1.06–1.15)*	1.14 (1.09–1.20)*
Number of PCG^a^ (ref: no)				
1	Colo	1.36 (1.31–1.42)*	1.28 (1.23–1.33)*	1.27 (1.23–1.32)*
	FOBT	1.51 (1.43–1.59)*	1.46 (1.37–1.55)*	1.40 (1.31–1.49)*
2	Colo	1.44 (1.37–1.50)*	1.35 (1.29–1.40)*	1.38 (1.33–1.44)*
	FOBT	1.58 (1.50–1.67)*	1.61 (1.51–1.71)*	1.48 (1.38–1.58)*
≥3	Colo	1.69 (1.63–1.77)*	1.50 (1.45–1.56)*	1.50 (1.45–1.55)*
	FOBT	1.52 (1.44–1.60)*	1.61 (1.52–1.71)*	1.63 (1.53–1.73)*

a Pharmacy-based Cost Group [26], based on data of the previous year.

*Confidence intervals do not cross the OR of 1.

All the odds ratios are adjusted for the other covariates, in a multinomial logistic regression model.

CHF, Swiss Franc; FOBT, fecal occult blood test.

We found a significant interaction of age on the association between years and overall CRC testing (*p* < 0.01, Figure 2). Over the years, the odds ratios of being tested contrasting the younger and older insurees (50–69 vs. 70–75 year olds) decreased. There was also a significant interaction of deductible on the association between years and overall CRC testing (*p* < 0.01, Figure 2). Over the years, the odds ratio between lower and higher deductible categories decreased.

## Discussion

This study found that the yearly claimed CRC testing rate has been rising in Switzerland from 8.1% in 2012 to 9.9% in 2018, due to an increase in the colonoscopy rate (from 5.0 to 7.6%). After the new reimbursment policy went into effect in 2013, the amount of reimbursement claim rose more in the 50-69-year-old than in the 70-75-year-old age category, leading to a decrease in difference in testing rates between age categories. Insurees with HDHP had lower claimed CRC testing rates in 2012 compared to insurees with LDHL and continued to do so in 2018. Even if the relative difference between healthcare plans shrank from 2012 to 2018, the absolute difference in CRC testing rate between healthcare plans increased from 2012 to 2018, in particular for colonoscopy.

Our results are consistent with previous studies, which showed a steady increase of CRC testing rate in Switzerland from 2007 to 2014, with an increasing use of colonoscopy and a decrease in FOBT rate [9–12]. We confirm the associations between LDHP and increasing age with higher CRC testing rate [9–12, 32]. The participation in a managed-care model was also previously found to be associated with higher CRC testing rate [12]. Compared to the testing rate self-reported in the 2012 Swiss Health Interview Survey (SHIS) [9], we found a lower testing rate for every test, especially for FOBT (Supplementary File S2).

The data analyzed until 2018 represents the first attempt to monitor the yearly CRC testing rate in Switzerland to determine the potential effects on CRC testing of the 2013 policy change using claims data from health insurances. This change in law introduced the reimbursement of CRC screening test for people aged 50–69 years old. Reimbursement for CRC testing increased more among 50-69-year-olds than in those aged 70–75, which likely indicates CRC tests were becoming more common in this first age category, but we could not differentiate the continuation of the trend observed in the past years of an effect of the law. Though Swiss lawmakers stop reimbursements after age 69, most international guidelines recommend CRC screening until at least 75 [5, 33, 34]. We found that 70-75-year-olds are still tested for CRC, probably because Swiss healthcare professional follow these recommendations. This highlights the importance of reimbursement decisions on CRC screening rates.

Over the last decades, the financial burden of health insurance premium continuously increased in Switzerland and an increasing amount of insurees opt to HDHP or managed-care model, which offer reduced premiums. We found HDHP to be strongly correlated with lower claim for reimbursement and the difference persisted after the 2013 change in reimbursement policy, indicating that financial considerations might represent a barrier to being tested for CRC. The difference between LDHP and HDHP insurees might also simply reflect individual preferences for consumption of care with those opting for HDHP less likely to be interested in CRC screening. A person with LDHP might also be more likely to have other health conditions leading to diagnostic CRC testing and more access to healthcare providers who would offer the possibility of CRC screening. Evidence about the impact of cost-sharing models on CRC screening rates is still limited: the US Affordable Care Act (ACA) supressed cost-sharing for preventive screening in 2010, but studies either found no change in the rate of colonoscopy [22], or that no-charge screening was associated with a greater increase in colonoscopy rates among those with HDHP (≥$1000) than those with LDHP (≤$500) [23]. Future studies, in particular datasets with detailed information about reason for CRC testing (screening vs. diagnostic), should explore the effect of deductible on CRC screening rate. In particular if removing deductible and copay might lead to reduction in differences in CRC testing rates by healthcare plan.

### Limitations and Strengths

Our study had several limitations. First, because we could not differentiate between screening and diagnostic tests we were unable to specifically measure the change in CRC screening rates associated with the 2013 policy reimbursement law. The testing rates reported include tests performed for diagnostic and screening reasons. Second, we urge for careful interpretation of the data on FOBT. The dataset only contains data for bills submitted for reimbursement; we used these to estimate the real testing rate. The 2013 reimbursement policy may have increase the number of bills submitted without actually increasing the true testing rate. When comparing the rate of colonoscopies or both tests performed in the last year in the 2012 SHIS data, which relies on self-report, we found one to two times higher colonoscopy rates that in the Helsana dataset (Supplementary File S2). Because of the cost of a colonoscopy, insurees or gastroenterologists directly probably send most of the bills to the insurance and we likely catched most colonoscopies which were done. While some insurees might not have sent their bills, another explanation might be overreporting of colonosopies in the SHIS [35]. However, we found one to four time lower FOBT rates in the Helsana dataset compared to the SHIS dataset, especially by those with HDHP (Supplementary File S2). Even if we can posit some might be explained by overreporting bias in SHIS, an underreporting of FOBT in the Helsana dataset seems likely. Many insurees possibly renounce to claim reimbursement for small invoice, such as the FOBT, which remain below their deductible threshold and as such we do not capture these tests. Our analyses focused on the change in CRC testing over the years. So even if the dataset likely underreports the true FOBT rate, we do not expect that this source of bias changed over the years. A reduction in FOBT testing rate was also observed in the SHIS dataset. Third, since we were primarily interested in the change in testing rate over the years, we only analysed testing rate over each index year without tracking insurees over the 2012–2018 period. We did not determine the prevalence of those up-to-date with CRC testing (colonoscopy within the last 10 years or FOBT within last 2 years) each year, since our dataset was restricted to years 2012–2018. We have no data of the colonoscopies performed before 2012. Fourth, since data was only available from 2012 and the reimbursement reform occurred in 2013, we are inherently limited in carefully describing the trends before 2013. Fifth, the significant interaction term for age on the association between years and overall CRC testing is primarily driven by a smaller decrease in FOBT use in younger populations; this may in part be due to people beginning screening with FOBT.

Our study also had several strengths. Our data was broadly representative of the Swiss population regardless of the cantons of residence. We had comprehensive information about insurees and no data was missing for the covariates we used. Our reliance on administrative data also enabled us to shorten the time between data collection and publication of the results.

### Conclusion

Current guidelines recommend patients be presented a menu of options for CRC screening tests and to be able to decide which test they want to undergo, according to their preferences and values [5, 6]. Besides individual factors, financial considerations should not represent a barrier to be screened. FOBT is inexpensive, innocuous and easy to use but underused in Switzerland, and could thus help to increase the CRC screening rate. However, insurees who prefer colonoscopy to FOBT should not face financial barriers to do so. Organized CRC screening programs, who waive deductible for both FOBT and colonoscopy in Switzerland, should help to understand whether this waiver contribute to further reduction in differences in CRC screening between insurees with HDHP and LDHP.

CRC testing rate in Switzerland increased from 2012 to 2018, in particular among Swiss insurees aged 50–69, the target population of the reimbursement law of 2013. We cannot determine if the increase can be imputed to the 2013 law or if the change reflects the continuation of the trend which started earlier than 2013. The rate of colonoscopy increased, while the rate of FOBT decreased. Those with a high-deductible health plan remain less likely to be tested for CRC. Future studies should explore whether encouraging more participants to opt for FOBT, and waiving the deductible may increase the overall CRC testing rate.
