# Supplementary material for: Change in Colorectal Cancer Tests Submitted for Reimbursement in Switzerland 2012–2018: Evidence from Claims Data of a Large Insurance
Source: Int J Public Health. 2021 Oct 28;66:1604073. doi: 10.3389/ijph.2021.1604073 (PMC8565273; doi:10.3389/ijph.2021.1604073)
Supplement: Supplementary file 1 [file DataSheet1.docx]

**Change in colorectal cancer tests submitted for reimbursement in Switzerland 2012-2018: Evidence from claims data of a large insurance**

# Appendix

Supplementary File 1: Billing codes used to identify the group colonoscopy and FOBT.

| **Catalogue** | **Code** | | | **Description** | **Comments** |
| --- | --- | --- | --- | --- | --- |
| **Colonoscopy** | |  | |  |  |
| Inpatient colonoscopy | |  | |  |  |
| CHOP | | | 45.23 | Colonoscopy |  |
| CHOP | | | 45.24 | Sigmoidoscopy |  |
| CHOP | | | 45.25 | Colonoscopy with biopsy |  |
| CHOP | | | 45.41.11 | Colonoscopy | only present in 2018 |
| CHOP | | | 45.41.15 | Colonoscopy | only present in 2018 |
| CHOP | | | 48.23 | Sigmoidoscopy |  |
| CHOP | | | 48.24 | Sigmoidoscopy |  |
| CHOP | | | 48.29.1% | Chromoendoscopy |  |
| CHOP | | | 48.29.2% | Endoscopic Laser-endomicroscopy |  |
| CHOP | | | 48.36 | Sigmoidoscopy |  |
| DRG | | | G48% | Colonoscopy DRG |  |
| Outpatient colonoscopy |  | | |  |  |
| TARMED | 19.06 | | | Colonoscopy |  |
| TARMED | 19.07 | | | Sigmoidoscopy |  |
| **FOBT** | |  | |  |  |
| AL | 1583.00 | | | FOBT |  |
| AL | 1583.01 | | | FOBT | only present in 2017 |

CHOP = Swiss Classification of Surgical Operation ; DRG = Swiss Diagnosis-Related Groups; TARMED = Swiss Ambulatory Procedures codes; AL = Swiss Analysis List of laboratory measures

Supplementary File 2: Comparison of declared testing rate from the Swiss Health Interview Survey (SHIS) and claimed testing rate from the Helsanas Database for FOBT, colonoscopy and both in 2012. The number in bracket is the ratio of the testing rates in SHS over these in Helsanas Database.

|  |  | SHIS 2012 | | | Helsana 2012 | | | Ratio SHIS/Helsana | | |
| --- | --- | --- | --- | --- | --- | --- | --- | --- | --- | --- |
| Test | Deductible | 50-59 | 60-69 | 70-75 | 50-59 | 60-69 | 70-75 | 50-59 | 60-69 | 70-75 |
| FOBT only | 300  (%, 95% CI) | 4.9% | 5.1% | 7.9% | 2.6% | 3.9% | 4.4% | 1.9 | 1.3 | 1.8 |
|  |  | (3.6 – 6.7) | (4.0 – 6.6) | (5.9 – 10.5) | (2.5 -2.8) | (3.7 – 4.0) | (4.2 – 4.6) |  |  |  |
|  | 500 – 1500  (%, 95% CI) | 4.0% | 4.9% | 7.0% | 2.2% | 3.4% | 3.8% | 1.8 | 1.4 | 1.8 |
|  |  | (2.8 – 5.6) | (3.6 – 6.7) | (4.4 – 10.8) | (2.1 – 2.3) | (3.2 – 3.6) | (3.5 – 4.1) |  |  |  |
|  | 2000 – 2500  (%, 95% CI) | 3.9% | 5.9% | 11.3% | 1.3% | 2.2% | 2.6% | 2.9 | 2.7 | 4.4 |
|  |  | (2.2 – 6.6) | (3.4 – 9.9) | (5.2 – 23.1) | (1.2 – 1.5) | (1.9 – 2.5) | (2.1 – 3.2) |  |  |  |
| Colonoscopy only | 300  (%, 95% CI) | 5.0% | 8.4% | 4.5% | 5.1% | 6.6% | 6.7% | 1.0 | 1.3 | 0.7 |
|  |  | (3.7 – 6.7) | (6.6 – 10.6) | (3.1 – 6.4) | (4.9 – 5.3) | (6.4 – 6.8) | (6.4 – 6.9) |  |  |  |
|  | 500 – 1500  (%, 95% CI) | 4.5% | 6.8% | 6.5% | 4.0% | 5.4% | 6.0% | 1.1 | 1.3 | 1.1 |
|  |  | (3.2 – 6.5) | (5.1 – 8.9) | (4.2 – 10.0) | (3.9 – 4.2) | (5.2 – 5.6) | (5.7 – 6.3) |  |  |  |
|  | 2000 – 2500  (%, 95% CI) | 3.9% | 4.1% | 4.7% | 2.6% | 3.7% | 3.7% | 1.5 | 1.1 | 1.3 |
|  |  | (2.2 – 6.8) | (2.4 – 7.1) | (1.4 – 14.7) | (2.4 – 2.8) | (3.3 – 4.0) | (3.1 – 4.4) |  |  |  |
| Colo+FOBT | 300  (%, 95% CI) | 11.5% | 15.3% | 13.7% | 7.5% | 10.1% | 10.6% | 1.5 | 1.5 | 1.3 |
|  |  | (9.3 – 14.1) | (13.1 – 17.9) | (11.1 – 16.8) | (7.3 – 7.7) | (9.9 – 10.3) | (10.3 – 10.9) |  |  |  |
|  | 500 – 1500  (%, 95% CI) | 9.0% | 12.9% | 15.8% | 6.0% | 8.4% | 9.4% | 1.5 | 1.5 | 1.7 |
|  |  | (7.1 – 11.3) | (10.6 – 15.6) | (12.0 – 20.5) | (5.8 – 6.1) | (8.1 – 8.6) | (9.0 – 9.8) |  |  |  |
|  | 2000 – 2500  (%, 95% CI) | 8.0% | 10.2% | 16.2% | 3.8% | 5.6% | 6.0% | 2.1 | 1.8 | 2.7 |
|  |  | (5.4 – 11.6) | (6.9 – 14.7) | (8.5 – 28.6) | (3.6 – 4.1) | (5.2 – 6.0) | (5.2 – 6.7) |  |  |  |

Supplementary File 3: Proportion of 50-75-year old insurees with colonoscopy or FOBT billing data each year from 2012 to 2018, Helsana Database.

| **Variable** | **Colonoscopy** | | | | | | | **FOBT/both** | | | | | | |
| --- | --- | --- | --- | --- | --- | --- | --- | --- | --- | --- | --- | --- | --- | --- |
|  | **2012** | **2013** | **2014** | **2015** | **2016** | **2017** | **2018** | **2012** | **2013** | **2014** | **2015** | **2016** | **2017** | **2018** |
| Population  N, %, (95% CI) | 17 630 | 20 619 | 22 717 | 24 110 | 25 886 | 24 594 | 26 483 | 11 101 | 10 654 | 9 897 | 9 016 | 8 756 | 8 073 | 7 895 |
|  | 5.0 | 5.8 | 6.3 | 6.7 | 7.3 | 7.2 | 7.6 | 3.1 | 3.0 | 2.8 | 2.5 | 2.50 | 2.40 | 2.3 |
|  | (4.9-5.0) | (5.7-5.8) | (6.2-6.4) | (6.6-6.8) | (7.2-7.4) | (7.1-7.3) | (7.5-7.7) | (3.1-3.2) | (2.9-3.0) | (2.7-2.8) | (2.5-2.6) | (2.4 –2.5) | (2.3 –2.4) | (2.2 –2.3) |
| **Age in years** |  |  |  |  |  |  |  |  |  |  |  |  |  |  |
| 50-69  N, %, (95% CI) | 13 754 | 15 949 | 17 786 | 18 622 | 20 000 | 18 937 | 20 482 | 8 342 | 7 831 | 7 244 | 6 570 | 6 327 | 5 814 | 5 722 |
|  | 4.8 | 5.5 | 6.1 | 6.5 | 7.1 | 7.0 | 7.4 | 2.9 | 2.7 | 2.5 | 2.3 | 2.2 | 2.1 | 2.1 |
|  | (4.7-4.8) | (5.4-5.6) | (6.1-6.2) | (6.4-6.5) | (7.0-7.2) | (6.9-7.1) | (7.3-7.5) | (2.8-2.9) | (2.6-2.8) | (2.5-2.6) | (2.2-2.3) | (2.2-2.3) | (2.1-2.2) | (2.0-2.1) |
| 70-75 N, %, (95% CI) | 3 876 | 4 670 | 4 931 | 5 488 | 5 886 | 5 657 | 6 001 | 2 759 | 2 823 | 2 653 | 2 446 | 2 429 | 2 259 | 2 173 |
|  | 5.8 | 6.8 | 7.0 | 7.7 | 8.1 | 7.9 | 8.3 | 4.1 | 4.1 | 3.8 | 3.4 | 3.40 | 3.10 | 3.0 |
|  | (5.6-6.0) | (6.6-7.0) | (6.8-7.2) | (7.5-7.9) | (7.9-8.3) | (7.7-8.1) | (8.1-8.5) | (4.0-4.3) | (4.0-4.3) | (3.6-3.9) | (3.3-3.6) | (3.2 –3.5) | (3.0 –3.3) | (2.9 –3.1) |
| **Gender** |  |  |  |  |  |  |  |  |  |  |  |  |  |  |
| Female N, %, (95% CI) | 9 356 | 10 907 | 11 850 | 12 655 | 13 477 | 12 803 | 13 700 | 5 479 | 5 255 | 4 935 | 4 472 | 4 417 | 4 168 | 4 091 |
|  | 5.0 | 5.8 | 6.3 | 6.7 | 7.3 | 7.1 | 7.5 | 2.9 | 2.8 | 2.6 | 2.4 | 2.40 | 2.30 | 2.2 |
|  | (4.9-5.1) | (5.7-5.9) | (6.2-6.4) | (6.6-6.8) | (7.1-7.4) | (7.0-7.3) | (7.4-7.7) | (2.9-3.0) | (2.7-2.9) | (2.6-2.7) | (2.3-2.4) | (2.3 –2.5) | (2.3 –2.4) | (2.2 –2.3) |
| Male N, %, (95% CI) | 8 274 | 9 712 | 10 867 | 11 455 | 12 409 | 11 791 | 12 783 | 5 622 | 5 399 | 4 962 | 4 544 | 4 339 | 3 905 | 3 804 |
|  | 4.9 | 5.7 | 6.3 | 6.7 | 7.3 | 7.2 | 7.7 | 3.3 | 3.2 | 2.9 | 2.6 | 2.60 | 2.40 | 2.3 |
|  | (4.8-5.0) | (5.6-5.8) | (6.2-6.5) | (6.5-6.8) | (7.2-7.5) | (7.1-7.3) | (7.5-7.8) | (3.2-3.4) | (3.1-3.2) | (2.8-3.0) | (2.6-2.7) | (2.5 –2.6) | (2.3 –2.5) | (2.2 –2.4) |
| **Residence** |  |  |  |  |  |  |  |  |  |  |  |  |  |  |
| Urban N, %, (95% CI) | 4 824 | 5 680 | 6 182 | 6 712 | 7 021 | 6 664 | 7 296 | 2 881 | 2 785 | 2 500 | 2 246 | 2 180 | 1 988 | 2 082 |
|  | 5.0 | 5.8 | 6.3 | 6.9 | 7.3 | 7.2 | 7.6 | 3.0 | 2.8 | 2.6 | 2.3 | 2.3 | 2.2 | 2.2 |
|  | (4.8-5.1) | (5.6-6.0) | (6.2-6.5) | (6.7-7.0) | (7.2-7.5) | (7.1-7.4) | (7.4-7.8) | (2.9-3.1) | (2.7-3.0) | (2.5-2.7) | (2.2-2.4) | (2.2 –2.4) | (2.1 –2.3) | (2.1 –2.3) |
| Intermediate N, %, (95% CI) | 9 059 | 10 581 | 11 763 | 12 479 | 13 295 | 12 622 | 13 365 | 5 752 | 5 538 | 5 196 | 4 716 | 4 577 | 4 244 | 3 931 |
|  | 5.2 | 6.0 | 6.7 | 7.0 | 7.6 | 7.5 | 7.9 | 3.3 | 3.2 | 2.9 | 2.7 | 2.6 | 2.5 | 2.3 |
|  | (5.1-5.3) | (5.9-6.1) | (6.5-6.8) | (6.9-7.2) | (7.5-7.8) | (7.3-7.6) | (7.8-8.0) | (3.2-3.4) | (3.1-3.2) | (2.9-3.0) | (2.6-2.7) | (2.6 –2.7) | (2.4 –2.6) | (2.3 –2.4) |
| Rural N, %, (95% CI) | 3 747 | 4 358 | 4 772 | 4 919 | 5 570 | 5 308 | 5 822 | 2 468 | 2 331 | 2 201 | 2 054 | 1 999 | 1 841 | 1 882 |
|  | 4.4 | 5.1 | 5.6 | 5.8 | 6.6 | 6.5 | 7.0 | 2.9 | 2.7 | 2.6 | 2.4 | 2.4 | 2.2 | 2.2 |
|  | (4.3-4.6) | (5.0-5.3) | (5.4-5.7) | (5.6-5.9) | (6.4-6.7) | (6.3-6.6) | (6.8-7.1) | (2.8-3.0) | (2.6-2.9) | (2.5-2.7) | (2.3-2.5) | (2.3 –2.5) | (2.1 –2.3) | (2.1 –2.4) |
| **Deductible (CHF)** |  |  |  |  |  |  |  |  |  |  |  |  |  |  |
| 300 N, %, (95% CI) | 10 800 | 12 736 | 13 908 | 15 121 | 16 177 | 15 374 | 16 441 | 6 748 | 6 538 | 6 178 | 5 588 | 5 484 | 4 978 | 4 902 |
|  | 5.7 | 6.6 | 7.1 | 7.7 | 8.3 | 8.0 | 8.5 | 3.6 | 3.4 | 3.2 | 2.8 | 2.8 | 2.6 | 2.5 |
|  | (5.6-5.8) | (6.5-6.7) | (7.0-7.2) | (7.5-7.8) | (8.1-8.4) | (7.9-8.2) | (8.4-8.7) | (3.5-3.6) | (3.3-3.5) | (3.1-3.2) | (2.8-2.9) | (2.7 –2.9) | (2.5 –2.7) | (2.5 –2.6) |
| 500 N, %, (95% CI) | 3 965 | 4 511 | 4 785 | 4 752 | 4 979 | 4 708 | 4 840 | 2 601 | 2 446 | 2 112 | 1 911 | 1 832 | 1 664 | 1 626 |
|  | 4.9 | 5.8 | 6.4 | 6.6 | 7.2 | 7.3 | 7.7 | 3.2 | 3.1 | 2.8 | 2.6 | 2.7 | 2.6 | 2.6 |
|  | (4.8-5.1) | (5.6-6.0) | (6.2-6.5) | (6.4-6.7) | (7.0-7.4) | (7.1-7.5) | (7.5-8.0) | (3.1-3.4) | (3.0-3.3) | (2.7-2.9) | (2.5-2.8) | (2.5 –2.8) | (2.5 –2.7) | (2.5 –2.7) |
| 1000/1500 N, %, (95% CI) | 1 736 | 1 914 | 2 171 | 2 183 | 2 325 | 2 144 | 2 291 | 1 065 | 960 | 863 | 791 | 717 | 698 | 610 |
|  | 3.8 | 4.3 | 5.0 | 5.2 | 5.8 | 5.8 | 6.3 | 2.3 | 2.2 | 2.0 | 1.9 | 1.8 | 1.9 | 1.7 |
|  | (3.6-4.0) | (4.1-4.5) | (4.8-5.2) | (5.0-5.4) | (5.6-6.0) | (5.5-6.0) | (6.1-6.6) | (2.2-2.5) | (2.0-2.3) | (1.9-2.1) | (1.8-2.0) | (1.7 –1.9) | (1.7 –2.0) | (1.5 –1.8) |
| 2000/2500 N, %, (95% CI) | 1 129 | 1 458 | 1 853 | 2 054 | 2 405 | 2 368 | 2 911 | 687 | 710 | 744 | 726 | 723 | 733 | 757 |
|  | 2.9 | 3.3 | 4.0 | 4.2 | 4.8 | 4.7 | 5.1 | 1.8 | 1.6 | 1.6 | 1.5 | 1.5 | 1.5 | 1.3 |
|  | (2.7-3.1) | (3.2-3.5) | (3.9-4.2) | (4.1-4.4) | (4.6-5.0) | (4.5-4.9) | (4.9-5.3) | (1.6-1.9) | (1.5-1.8) | (1.5-1.7) | (1.4-1.6) | (1.4 –1.6) | (1.4 –1.6) | (1.2 –1.4) |
| **Managed care** |  |  |  |  |  |  |  |  |  |  |  |  |  |  |
| No N, %, (95% CI) | 10 098 | 11 091 | 11 298 | 11 205 | 11 416 | 10 328 | 10 286 | 6 126 | 5 536 | 4 789 | 4 117 | 3 779 | 3 316 | 2 976 |
|  | 5.0 | 5.8 | 6.3 | 6.7 | 7.3 | 7.2 | 7.6 | 3.1 | 2.9 | 2.7 | 2.5 | 2.4 | 2.3 | 2.2 |
|  | (4.9-5.1) | (5.7-5.9) | (6.2-6.4) | (6.6-6.9) | (7.2-7.5) | (7.0-7.3) | (7.4-7.7) | (3.0-3.1) | (2.8-3.0) | (2.6-2.7) | (2.4-2.6) | (2.4 –2.5) | (2.2 –2.4) | (2.1 –2.3) |
| Yes N, %, (95% CI) | 7 532 | 9 528 | 11 419 | 12 905 | 14 470 | 14 266 | 16 197 | 4 975 | 5 118 | 5 108 | 4 899 | 4 977 | 4 757 | 4 919 |
|  | 4.9 | 5.7 | 6.3 | 6.7 | 7.3 | 7.2 | 7.6 | 3.2 | 3.0 | 2.8 | 2.5 | 2.5 | 2.4 | 2.3 |
|  | (4.8-5.0) | (5.6-5.8) | (6.2-6.5) | (6.5-6.8) | (7.1-7.4) | (7.0-7.3) | (7.5-7.7) | (3.1-3.3) | (3.0-3.1) | (2.8-2.9) | (2.5-2.6) | (2.4 –2.6) | (2.3 –2.5) | (2.2 –2.4) |
| **Number of PCG^1^** |  |  |  |  |  |  |  |  |  |  |  |  |  |  |
| None N, %, (95% CI) | 5 823 | 6 356 | 7 086 | 7 960 | 8 429 | 7 390 | 8 409 | 3 572 | 3 159 | 2 884 | 2 705 | 2 600 | 2 226 | 2 279 |
|  | 3.7 | 4.2 | 4.8 | 5.2 | 5.7 | 5.4 | 5.9 | 2.3 | 2.1 | 1.9 | 1.8 | 1.8 | 1.6 | 1.6 |
|  | (3.6-3.8) | (4.1-4.3) | (4.7-4.9) | (5.1-5.3) | (5.6-5.8) | (5.3-5.6) | (5.8-6.0) | (2.2-2.3) | (2.0-2.2) | (1.9-2.0) | (1.7-1.8) | (1.7 –1.8) | (1.6 –1.7) | (1.5 –1.7) |
| 1 N, %, (95% CI) | 3 658 | 4 197 | 4 583 | 4 684 | 5 107 | 5 047 | 5 222 | 2 508 | 2 346 | 2 073 | 1 830 | 1 703 | 1 657 | 1 608 |
|  | 5.3 | 6.0 | 6.7 | 7.0 | 7.6 | 7.6 | 7.9 | 3.6 | 3.4 | 3.0 | 2.7 | 2.5 | 2.5 | 2.4 |
|  | (5.1-5.5) | (5.8-6.2) | (6.5-6.9) | (6.8-7.2) | (7.4-7.8) | (7.4-7.8) | (7.6-8.1) | (3.5-3.8) | (3.2-3.5) | (2.9-3.1) | (2.6-2.9) | (2.4 –2.7) | (2.4 –2.6) | (2.3 –2.5) |
| 2 N, %, (95% CI) | 3 126 | 3 720 | 3 909 | 4 124 | 4 401 | 4 277 | 4 648 | 2 178 | 2 129 | 1 961 | 1 719 | 1 670 | 1 533 | 1 421 |
|  | 5.7 | 6.6 | 7.0 | 7.6 | 8.1 | 8.0 | 8.7 | 4.0 | 3.8 | 3.5 | 3.1 | 3.1 | 2.9 | 2.7 |
|  | (5.5-5.9) | (6.4-6.9) | (6.8-7.2) | (7.3-7.8) | (7.9-8.4) | (7.7-8.2) | (8.4-8.9) | (3.8-4.1) | (3.6-4.0) | (3.4-3.7) | (3.0-3.3) | (2.9 –3.2) | (2.7 –3.0) | (2.5 –2.8) |
| ≥3 N, %, (95% CI) | 5 023 | 6 346 | 7 139 | 7 342 | 7 949 | 7 880 | 8 204 | 2 843 | 3 020 | 2 979 | 2 762 | 2 783 | 2 657 | 2 587 |
|  | 6.8 | 7.8 | 8.3 | 8.6 | 9.2 | 9.0 | 9.5 | 3.9 | 3.7 | 3.5 | 3.2 | 3.2 | 3.0 | 3.0 |
|  | (6.6-7.0) | (7.6-8.0) | (8.1-8.5) | (8.4-8.8) | (9.0-9.4) | (8.8-9.2) | (9.3-9.7) | (3.7-4.0) | (3.6-3.9) | (3.3-3.6) | (3.1-3.3) | (3.1 –3.3) | (2.9 –3.2) | (2.9 –3.1) |

^1^ Pharmacy-based Cost Group [26], based on data of the previous year
Percentages refer to the proportion of insurees which were tested in the subgroup of the studied population, with the 95% confidence interval. CHF = Swiss Franc.

Supplementary File 4: Adjusted odd ratios of 50-75-year old insurees who underwent any CRC test, Helsana Database, 2012 to 2018

|  | **2012** | **2013** | **2014** | **2015** | **2016** | **2017** | **2018** |
| --- | --- | --- | --- | --- | --- | --- | --- |
|  | OR (CI 95%) | OR (CI 95%) | OR (CI 95%) | OR (CI 95%) | OR (CI 95%) | OR (CI 95%) | OR (CI 95%) |
| Age group (ref: 50-69) |  |  |  |  |  |  |  |
| 70-75 | 1.16 (1.13 – 1.20)* | 1.19 (1.16 – 1.22)* | 1.11 (1.08 – 1.15)* | 1.14 (1.11 – 1.17)* | 1.11 (1.08 – 1.14)* | 1.08 (1.05 – 1.11)* | 1.05 (1.02 – 1.08)* |
| Gender (ref: male) |  |  |  |  |  |  |  |
| Female | 0.92 (0.90 – 0.94) | 0.92 (0.90 – 0.94) | 0.92 (0.90 – 0.94) | 0.93 (0.91 – 0.95) | 0.93 (0.91 – 0.95) | 0.94 (0.92 – 0.96) | 0.93 (0.91 – 0.96) |
| Residence (ref: urban) |  |  |  |  |  |  |  |
| intermediate | 1.09 (1.06 – 1.13)* | 1.09 (1.06 – 1.12)* | 1.10 (1.07 – 1.13)* | 1.08 (1.05 – 1.11)* | 1.09 (1.06 – 1.12)* | 1.09 (1.06 – 1.12)* | 1.07 (1.04 – 1.10)* |
| rural | 0.95 (0.92 – 0.98) | 0.93 (0.90 – 0.96) | 0.93 (0.90 – 0.96) | 0.90 (0.87 – 0.93) | 0.95 (0.92 – 0.98) | 0.94 (0.91 – 0.97) | 0.96 (0.93 – 0.99) |
| Deductible (CHF) (ref: 300) |  |  |  |  |  |  |  |
| 500 | 0.91 (0.89 – 0.94) | 0.93 (0.90 – 0.95) | 0.92 (0.89 – 0.94) | 0.90 (0.87 – 0.92) | 0.91 (0.89 – 0.94) | 0.96 (0.93 – 0.98) | 0.96 (0.93 – 0.98) |
| 1000/1500 | 0.75 (0.72 – 0.78) | 0.73 (0.70 – 0.77) | 0.77 (0.74 – 0.80) | 0.75 (0.72 – 0.78) | 0.76 (0.73 – 0.79) | 0.80 (0.77 – 0.84) | 0.76 (0.76 – 0.82) |
| 2000/2500 | 0.59 (0.56 – 0.62) | 0.59 (0.56 – 0.62) | 0.64 (0.61 – 0.67) | 0.63 (0.60 – 0.65) | 0.64 (0.62 – 0.67) | 0.67 (0.64 – 0.70) | 0.65 (0.63 – 0.68) |
| Managed care (ref: no) |  |  |  |  |  |  |  |
| Yes | 1.09 (1.06 – 1.12)* | 1.08 (1.06 – 1.12)* | 1.11 (1.08 – 1.13)* | 1.07 (1.05 – 1.10)* | 1.07 (1.04 – 1.09)* | 1.07 (1.05 – 1.10)* | 1.08 (1.06 – 1.11)* |
| Number of PCG^1^ (ref: no) |  |  |  |  |  |  |  |
| 1 | 1.42 (1.37 – 1.47)* | 1.40 (1.35 – 1.44)* | 1.38 (1.34 – 1.43)* | 1.32 (1.28 – 1.37)* | 1.30 (1.26 – 1.34)* | 1.37 (1.33 – 1.42)* | 1.30 (1.26 – 1.34)* |
| 2 | 1.49 (1.44 – 1.55)* | 1.51 (1.45 – 1.56)* | 1.46 (1.41 – 1.52)* | 1.41 (1.36 – 1.46)* | 1.39 (1.35 – 1.44)* | 1.43 (1.38 – 1.48)* | 1.40 (1.36 – 1.45)* |
| ≥3 | 1.63 (1.57 – 1.68)* | 1.64 (1.59 – 1.70)* | 1.62 (1.57 – 1.67)* | 1.53 (1.48 – 1.58)* | 1.52 (1.47 – 1.57)* | 1.58 (1.53 – 1.63)* | 1.53 (1.48 – 1.57)* |

^1^ Pharmacy-based Cost Group [26], based on data of the previous year

CHF = Swiss Franc.

All the odds ratios are adjusted for the others covariates, in a binomial logistic regression model.

* Confidence intervals do not cross the OR of 1.

Supplementary File **5**: **multinomial** logistic regression model, adjusted odd ratios of 50-75-year old insurees who underwent **colonoscopy or FOBT**, Helsana Database, 2012 to 2018

|  | **Test** | **2012** | **2013** | **2014** | **2015** | **2016** | **2017** | **2018** |
| --- | --- | --- | --- | --- | --- | --- | --- | --- |
|  |  | OR (CI 95%) | OR (CI 95%) | OR (CI 95%) | OR (CI 95%) | OR (CI 95%) | OR (CI 95%) | OR (CI 95%) |
| Age group (ref: 50-69) |  |  |  |  |  |  |  |  |
| 70-75 | Colo | 1.08 (1.04 – 1.12)* | 1.10 (1.06 – 1.14)* | 1.02 (0.99 – 1.05) | 1.07 (1.03 – 1.10)* | 1.03 (1.00 – 1.07) | 1.01 (0.98 – 1.04) | 0.99 (0.96 – 1.02) |
|  | FOBT | 1.30 (1.24 – 1.36)* | 1.38 (1.31 – 1.44)* | 1.35 (1.29 – 1.41)* | 1.34 (1.27 – 1.40)* | 1.33 (1.26 – 1.39)* | 1.30 (1.23 – 1.37)* | 1.26 (1.20 – 1.33)* |
| Gender (ref: male) |  |  |  |  |  |  |  |  |
| Female | Colo | 0.97 (0.94 – 1.00) | 0.97 (0.94 – 1.00) | 0.95 (0.92 – 0.97)* | 0.96 (0.93 – 0.98)* | 0.95 (0.92 – 0.97)* | 0.94 (0.92 – 0.97)* | 0.94 (0.91 – 0.96)* |
|  | FOBT | 0.84 (0.80 – 0.87)* | 0.84 (0.80 – 0.87)* | 0.86 (0.82 – 0.89)* | 0.85 (0.82 – 0.89)* | 0.88 (0.85 – 0.92)* | 0.93 (0.89 – 0.97)* | 0.93 (0.89 – 0.97)* |
| Residence (ref: urban) |  |  |  |  |  |  |  |  |
| intermediate | Colo | 1.07 (1.04 – 1.11)* | 1.07 (1.03 – 1.10)* | 1.08 (1.04 – 1.11)* | 1.05 (1.01 – 1.08) | 1.06 (1.03 – 1.10)* | 1.06 (1.03 – 1.09)* | 1.06 (1.03 – 1.09)* |
|  | FOBT | 1.13 (1.08 – 1.18)* | 1.13 (1.08 – 1.18)* | 1.17 (1.11 – 1.23)* | 1.17 (1.12 – 1.24)* | 1.18 (1.12 – 1.24)* | 1.19 (1.12 – 1.25)* | 1.09 (1.03 – 1.15)* |
| rural | Colo | 0.92 (0.88 – 0.96)* | 0.91 (0.87 – 0.94)* | 0.90 (0.86 – 0.93)* | 0.85 (0.82 – 0.88)* | 0.91 (0.88 – 0.95)* | 0.91 (0.88 – 0.94)* | 0.93 (0.90 – 0.97)* |
|  | FOBT | 1.00 (0.95 – 1.06) | 0.98 (0.93 – 1.04) | 1.02 (0.96 – 1.08) | 1.06 (1.00 – 1.12) | 1.06 (0.99 – 1.12) | 1.06 (0.99 – 1.13) | 1.06 (0.99 – 1.12) |
| Deductible (CHF) (ref: 300) |  |  |  |  |  |  |  |  |
| 500 | Colo | 0.90 (0.87 - 0.93)* | 0.91 (0.88 – 0.94)* | 0.92 (0.89 – 0.95)* | 0.87 (0.85 – 0.90)* | 0.89 (0.86 – 0.92)* | 0.93 (0.90 – 0.97)* | 0.93 (0.90 – 0.96)* |
|  | FOBT | 0.93 (0.89 – 0.98)* | 0.96 (0.91 – 1.00) | 0.91 (0.87 – 0.96)* | 0.96 (0.91 – 1.01) | 0.97 (0.92 – 1.03) | 1.03 (0.97 – 1.09) | 1.05 (1.00 – 1.12) |
| 1000/1500 | *Colo* | 0.76 (0.72 – 0.80)* | 0.74 (0.71 – 0.78)* | 0.79 (0.75 – 0.83)* | 0.75 (0.71 – 0.78)* | 0.77 (0.73 – 0.80)* | 0.97 (0.76 – 0.83)* | 0.80 (0.77 – 0.84)* |
|  | *FOBT* | 0.73 (0.68 – 0.78)* | 0.71 (0.67 – 0.77)* | 0.71 (0.66 – 0.77)* | 0.76 (0.70 – 0.82)* | 0.72 (0.67 – 0.79)* | 0.83 (0.77 – 0.91)* | 0.75 (0.68 – 0.81)* |
| 2000/2500 | Colo | 0.60 (0.56 - 0.64)* | 0.60 (0.57 – 0.64)* | 0.65 (0.62 – 0.69)* | 0.62 (0.59 – 0.65)* | 0.65 (0.62 – 0.68)* | 0.66 (0.63 – 0.70)* | 0.66 (0.63 – 0.69)* |
|  | FOBT | 0.57 (0.52 – 0.62)* | 0.56 (0.52 – 0.61)* | 0.60 (0.56 -0.66)* | 0.63 (0.58 – 0.69)* | 0.62 (0.57 – 0.67)* | 0.68 (0.63 – 0.74)* | 0.61 (0.57 – 0.67)* |
| Managed care (ref: no) |  |  |  |  |  |  |  |  |
| Yes | Colo | 1.06 (1.02 -1.09)* | 1.06 (1.03 – 1.09)* | 1.09 (1.06 – 1.12)* | 1.06 (1.03 – 1.09)* | 1.05 (1.03 – 1.08)* | 1.06 (1.03 – 1.09)* | 1.07 (1.04 – 1.10)* |
|  | FOBT | 1.15 (1.10 – 1.19)* | 1.14 (1.09 – 1.18)* | 1.15 (1.11 – 1.20)* | 1.11 (1.06 – 1.15)* | 1.11 (1.06 – 1.16)* | 1.11 (1.06 – 1.16)* | 1.14 (1.09 – 1.20)* |
| Number of PCG^1^ (ref: no) |  |  |  |  |  |  |  |  |
| 1 | Colo | 1.36 (1.31 – 1.42)* | 1.35 (1.30 – 1.41)* | 1.35 (1.30 – 1.41)* | 1.28 (1.23 – 1.33)* | 1.28 (1.24 – 1.33)* | 1.35 (1.30 -1.40)* | 1.27 (1.23 – 1.32)* |
|  | FOBT | 1.51 (1.43 – 1.59)* | 1.49 (1.41 – 1.57)* | 1.46 (1.37 – 1.54)* | 1.46 (1.37 – 1.55)* | 1.35 (1.27 – 1.44)* | 1.45 (1.36 – 1.55)* | 1.40 (1.31 – 1.49)* |
| 2 | Colo | 1.44 (1.37 – 1.50)* | 1.46 (1.39 – 1.52)* | 1.40 (1.34 -1.46)* | 1.35 (1.29 – 1.40)* | 1.34 (1.29 – 1.39)* | 1.38 (1.33 – 1.44)* | 1.38 (1.33 – 1.44)* |
|  | FOBT | 1.58 (1.50 – 1.67)* | 1.61 (1.52 – 1.70)* | 1.62 (1.53 – 1.72)* | 1.61 (1.51 – 1.71)* | 1.57 (1.47 – 1.67)* | 1.60 (1.49 – 1.71)* | 1.48 (1.38 – 1.58)* |
| ≥3 | Colo | 1.69 (1.63 – 1.77)* | 1.69 (1.63 – 1.76)* | 1.64 (1.58 – 1.70)* | 1.50 (1.45 – 1.56)* | 1.49 (1.44 – 1.55)* | 1.55 (1.50 – 1.61)* | 1.50 (1.45 – 1.55)* |
|  | FOBT | 1.52 (1.44 – 1.60)* | 1.55 (1.46 – 1.63)* | 1.56 (1.48 – 1.65)* | 1.61 (1.52 – 1.71)* | 1.60 (1.51 – 1.69)* | 1.67 (1.57 – 1.78)* | 1.63 (1.53 – 1.73)* |

^1^ Pharmacy-based Cost Group [26], based on data of the previous year

CHF = Swiss Franc.

All the odds ratios are adjusted for the others covariates, in a binomial logistic regression model.

* Confidence intervals do not cross the OR of 1
